# LncRNA XIST upregulates TRIM25 via negatively regulating miR-192 in hepatitis B virus-related hepatocellular carcinoma

**DOI:** 10.1186/s10020-021-00278-3

**Published:** 2021-04-15

**Authors:** Jiancheng Wang, Gang Yin, Hu Bian, Jiangli Yang, Pengcheng Zhou, Kai Yan, Cheng Liu, Pei Chen, Jun Zhu, Zhi Li, Tongqing Xue

**Affiliations:** 1 The People’s Hospital of Lianshui County, Huai’an City, 223400 Jiangsu Province People’s Republic of China; 2 Department of Intervention, The Second People’s Hospital of Huai’an City, Huai’an City, 223002 Jiangsu Province People’s Republic of China; 3 Department of Pain and Intervention, Huaiyin Hospital of Huai’an City, Huai’an City, 223300 Jiangsu Province People’s Republic of China; 4 Department of Interventional Radiology, Huaian Hospital of Huai’an City, No. 161 Zhenhuailou East Road, Huai’an City, 223200 Jiangsu Province People’s Republic of China; 5 The Third People’s Hospital of Yancheng City, No. 75 Juchang Road, Yancheng City, 224001 Jiangsu Province People’s Republic of China; 6 Department of Interventional Radiology, First Affiliated Hospital of Soochow University, No. 188 Shizi Street, Soochow City, 215006 Jiangsu Province People’s Republic of China

**Keywords:** Human hepatitis B virus, lncRNA XIST, Hepatocellular carcinoma, miR-192, TRIM25

## Abstract

**Background:**

Long non-coding RNA (lncRNA) XIST has been implicated in the progression of a variety of tumor diseases. The purpose of this study was to explore the molecular role of lncRNA XIST in human hepatitis B virus (HBV)-related hepatocellular carcinoma (HCC).

**Methods:**

The expression levels of lncRNA XIST, miR-192 and TRIM25 in HBV-related HCC tissues and HepG2.2.15 cells were detected by qRT-PCR. Biological information and luciferin gene reporter assay were performed to detect the interaction among lncRNA XIST, miR-192 and TRIM25. CCk-8 assay, wound healing assay and colony formation assay were conducted to detect the proliferation and migration ability of HepG2.2.15 cells.

**Results:**

qRT-PCR results showed that the expression levels of lncRNA XIST were remarkably increased in HBV-related HCC tissues and HepG2.2.15 cells. In addition, miR-192 was a direct target gene of lncRNA XIST, and the expression of miR-192 and lncRNA XIST were negatively correlated. Moreover, overexpression of miR-192 observably inhibited the proliferation and migration of HCC cells, while overexpression of lncRNA XIST showed an opposite effect. Furthermore, TRIM25 was a direct target of miR-192, and lncRNA XIST could up-regulate the expression of TRIM25 by targeting miR-192.

**Conclusion:**

LncRNA XIST could up-regulate the expression of TRIM25 by targeting and binding to miR-192, thus accelerating the occurrence and development of HCC.

## Background

Hepatocellular carcinoma (HCC) accounts for more than 90% of primary liver cancer (PLC) and is one of the most common malignant tumours with increasing morbidity and mortality (Ayuso et al. 2018a, b; Younossi et al. 2015). Currently, the most effective treatment for HCC is mainly surgical resection and transplantation, which still has high recurrence rate and poor prognosis (Galle et al. 2018). The occurrence of HCC is a complicated multi-factor and multi-stage process, which is related to many risk factors (El-Khoueiry et al. 2015). At present, HCC has been proven to be induced by inflammation, and more than 80% of HCC patients in China are associated with chronic hepatitis B virus (HBV) infection (de Martel et al. 2015; Papatheodoridis et al. 2015). The progression of HBV infection is hepatitis, fibrosis, cirrhosis, and hepatocellular carcinoma (Petruzziello 2018). Therefore, chronic HBV infection is a globally recognized major risk factor for HCC and can accelerate the progression of HCC liver failure (Saitta et al. 2015). Studies have found that proliferation and metastasis of tumor cells are one of the main reasons for the high mortality and poor prognosis of HCC (Fang et al. 2015). Therefore, finding targets related to tumor cell proliferation and metastasis has important clinical significance.

Long non-coding RNAs (lncRNAs) are a class of non-coding RNA molecules with a length greater than 200 bp (Quinn and Chang 2016). Recent studies have shown that lncRNAs are not only involved in the regulation of physiological processes such as chromosome remodeling, gene transcription and protein translation, but also related to the occurrence and development of various diseases such as tumors (Engreitz et al. 2016; Hanly et al. 2018; Yan et al. 2015). X chromosomal inactivating gene (XIST) is a lncRNA that has been shown to play a role in promoting or preventing cancer in different types of tumor disease (Yao et al. 2015). Studies have found that lncRNA XIST is abnormally expressed in multiple tumor tissues including non-small-cell lung cancer, glioblastoma, gastric cancer, hepatocellular carcinoma, ovarian cancer, and breast cancer (Li Chang et al. 2018b; Ma et al. 2017; Song et al. 2016; Xiong et al. 2017). Studies have confirmed that lncRNAs can competitively bind to microRNAs (miRNAs) as endogenous RNA (ceRNA), thus regulating the expression of miRNAs on downstream target genes (Zhou et al. 2016). MiRNAs are a class of endogenous non-coding single-stranded small molecule RNA with a length of about 19–22 nucleotides that involved in various physiological and pathological processes such as cell proliferation, differentiation, and the occurrence and development of cancer (Chiu et al. 2015; Thomson and Dinger 2016). Studies have found that miR-192 is mainly expressed in liver, kidney, colon and other tissues, and is downregulated in tumor tissues such as liver cancer, renal cell carcinoma and colon cancer, therefore is considered as a kind of miRNA with anti-cancer effect (Ast et al. 2018; Lian et al. 2016; Wu et al. 2016). MiR-192 was liver-abundant and specific and markedly downregulated in 5 type cancer stem cells (CSC) from HCC samples (Gu et al. 2019). It was reported that LncRNA XIST promoted the progression of colorectal cancer via the miR-192-5p/EIF5A2 axis (Zhao et al. 2020). Our bioinformatic analysis results showed that miR-192 contained the potential binding sites of lncRNA XIST.

Mature miRNAs can degrade or inhibit mRNA translation by completely or incompletely pairing with the 3′-UTR region of mRNA to exert their physiological functions (Lu et al. 2015). Ubiquitin ligase TRIM25, belonging to the tripartite motif (TRIM) family proteins, is a transcription factor that regulates the occurrence and development of a variety of diseases through ubiquitination or ubiquitination (Lee et al. 2018). Studies have found that TRIM25 is involved in the development of prostate cancer, endometrial cancer, ovarian cancer, breast cancer and other cancers (Takayama et al. 2018; Walsh et al. 2017; Zhu et al. 2016). Our bioinformatic analysis using microRNA.org software found that TRIM25 contained the potential binding sites of miR-192. However, the roles of lncRNA XIST, miR-192 and TRIM25 in HBV-related HCC are unclear. In this study, human HBV-related HCC tissues and HepG2.2.15 cells were used as research objects to explore the molecular mechanism of lncRNA XIST, miR-192, and TRIM25 in HBV-related HCC, aiming to provide a theoretical basis for targeted treatment of HBV-related HCC.

## Materials and methods

### Tissues collection

Liver tissues and adjacent HBV-related HCC tissues were obtained from 50 patients undergoing HCC resection in Huai’an Hospital of Huai’an (NO. 2020007). This study was approved by the Ethics Committee of aforementioned hospital. All collected specimens were immediately frozen in liquid nitrogen and then stored at − 80 °C before use.

### Cells

HepG2.2.15 cells transfected by HBV and HepG2 cells were used as subject cells. Cells were cultured in RPMI 1640 medium (Gibco BRL) containing 10% FBS (Gibco BRL). Negative control (NC) siRNA and XIST siRNA were purchased from Thermo Fisher Scientific, Inc. NC and miR-192 mimic were purchased from Gene Pharma (Shanghai, China). Empty vector (p-emptor vector) and lncRNA XIST over-expression vector (p-XIST) were purchased from Shanghai Jima Gene Co., Ltd. (Shanghai, China). These oligonucleotides or plasmids were transfected into HepG2.2.15 or HepG2 cells using Lipofectamine 2000 (Invitrogen, Carlsbad, CA, US).

### qRT-PCR assay

Total RNAs in tissues and cells were extracted using Trizol reagent (Invitrogen). SYBR green qPCR was conducted to detect the amplification of each gene. U6 was used as the internal reference for the expression of lncRNA and miRNA, and GAPDH was used as the internal reference for the expression of mRNA. The relative expression levels of gene were calculated using the 2^−ΔΔCT^ method. The primer sequences were shown in Table 1.

**Table 1:** The sequences of specific primers

Gene name	Primer sequence (5′ to 3′)
XIST	Forward: 5′-ACG CTG CAT GTG TCC TTA G-3′ Reverse: 5′-GAG CCT CTT ATA GCT GTT TG-3′
miR-192	Forward: 5′-CTGACCTATGAATTGACAGCCA-3′ Reverse: 5′-GCTGTCAACGATACGCTACGT-3′
TRIM25	Forward: 5′-GTCTCTACC CAGA ACAGTTTCC-3′ Reverse: 5′-ATCCAACACAGGCTGATTCC-3′
GAPDH	Forward: 5′-CACCCACTCCTCCACCTTTG-3′ Reverse: 5′-CCACCACC CTGTTGCTGTAG-3′
U6	Forward: 5′-TGCGGGTGCTCG CTT CGG CAGC-3′ Reverse: 5′-CCAGTGCAGGGTCCGAGGT-3′

### Luciferase reporter gene assay

LncRNA XIST-Mut or lncRNA XIST-Wt were co-transfected with miR-192 mimic or NC into HepG2.2.15 cells for 48 h. In addition, TRIM25-Mut or TRIM25-Wt was transfected with miR-192 mimic or NC into HepG2.2.15 cells for 48 h. Luciferase activity in cells was determined using the dual-luciferase assay kit (Promega) following the manufacturer’s instructions. *Renilla* luciferase was used as a control reporter for normalization.

### CCK-8 assay

Cells (5000 cells/well) were inoculated in 96-well culture plate for 24, 48, 72 and 96 h. Then CCK-8 (Dojindo, Japan) was used to determine the cell proliferation capacity following the manufacturer’s instructions.

### Wound healing experiment

When the cell layer was cultured to > 90% of the surface area of the culture dish, cell scratches were made using a 10 μL pipette head. Then the cells were cultured for another 48 h, and the degree of scratch closure was quantified.

### Colony formation assay

The transfected cells (1000 cells/well) were inoculated in a 6-well culture plate for 2 weeks. Cell colonies were then fixed with methanol and stained with methylene blue.

### Statistical analysis

All data were analyzed using SAS software (version 9.0; SAS Institute, Cary, NC, USA). Paired test was performed by grouping the 50 patients into high and low MCM3AP-AS1 level groups (n = 25) with the median expression level of lncRNA XIS***T*** in HCC as cutoff value. ANOVA (one-way) combined with Tukey test was used to compare multiple groups. Triple replicates were used in each experiments. All data were expressed as mean standard ± deviation (SD). Student's t test was used for significance analysis. A *P*-value < 0.05 was considered as significant.

## Results

### The expression of lncRNA XIST in HBV-related HCC tissues and cells

**Fig. 1:**
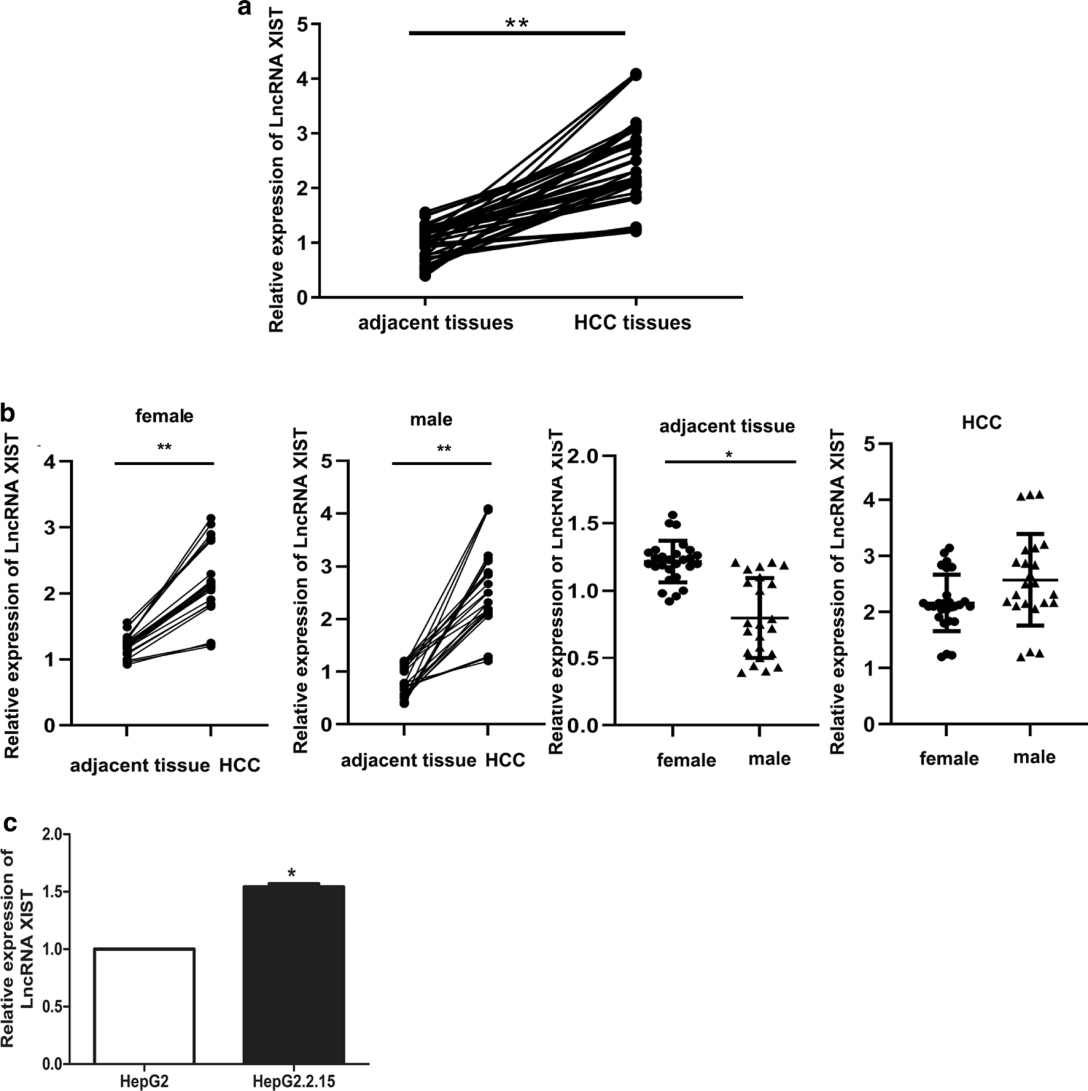
The expression of lncRNA XIST in HBV-related HCC tissues and adjacent cells. **a** Detection of the expression of lncRNA XIST in adjacent tissues (n = 50) and HCC tissues (n = 50). **b** Comparison of the expression of lncRNA XIST in gender subgroup (female = 27, male = 23) **c** Detection of the expression of lncRNA XIST in HepG2.2.15 and HepG2 cell lines. **P < 0.01, *P < 0.05

Firstly, the expression of lncRNA XIST in liver tissues (n = 50) and adjacent HBV-related HCC tissues (n = 50) were detected by qRT-PCR. The correlation between the expression of lnc XIST and clinical-pathological characteristic was shown in Table 2. The results showed that the expression of lncRNA XIST in HCC tissues was remarkably up-regulated compared with that in the adjacent tissues group (*P* < 0.01) (Fig. 1a). Since lncRNA XIST is regarded as an inactivator for X chromosome, it was speculated that its expression was correlated with patients’ gender. As shown in Fig. 1b, the expression of lncRNA XIST was higher in female adjacent tissues compared with that in male, but there was no difference in HCC tissue. In addition, the expression of lncRNA XIST was also measured in HepG2 and HepG2.2.15 cell lines. Compared with HepG2 cells, the expression of lncRNA XIST markedly increased in HepG2.2.15 cells (*P* < 0.05) (Fig. 1a). These results indicated that lncRNA XIST was highly expressed in HBV-related HCC tissues and cells.

**Table 2:** Correlation between Lnc XIST expressions and clinical-pathological characteristic in HCC (n = 50)

Parameters	Group	n	Lnc XIST expression		P level
			High (n = 25)	Low (n = 25)	
Age (years)	≤ 50	20	13	7	0.322
	> 50	30	12	18	
Gender	Female	27	7	20	0.509
	Male	23	18	5	
TNM stage	T1–T2	23	15	8	0.1609
	T3–T4	27	10	17	
Serum AFP	< 200	8	5	3	0.695
(ng/ml)	> 200	42	20	22	
Clinical stage	I–II	25	16	9	0.002**
	III–IV	25	9	16	
Vascular invasion	Yes	22	15	7	0.022*
	No	28	10	18	
Differentiation	Well	5	3	2	0.5112
	Moderate	34	17	17	
	Poor	11	5	6	

*P < 0.05, **P < 0.01

### Identification of miR-192 as a target gene of lncRNA XIST

**Fig. 2:**
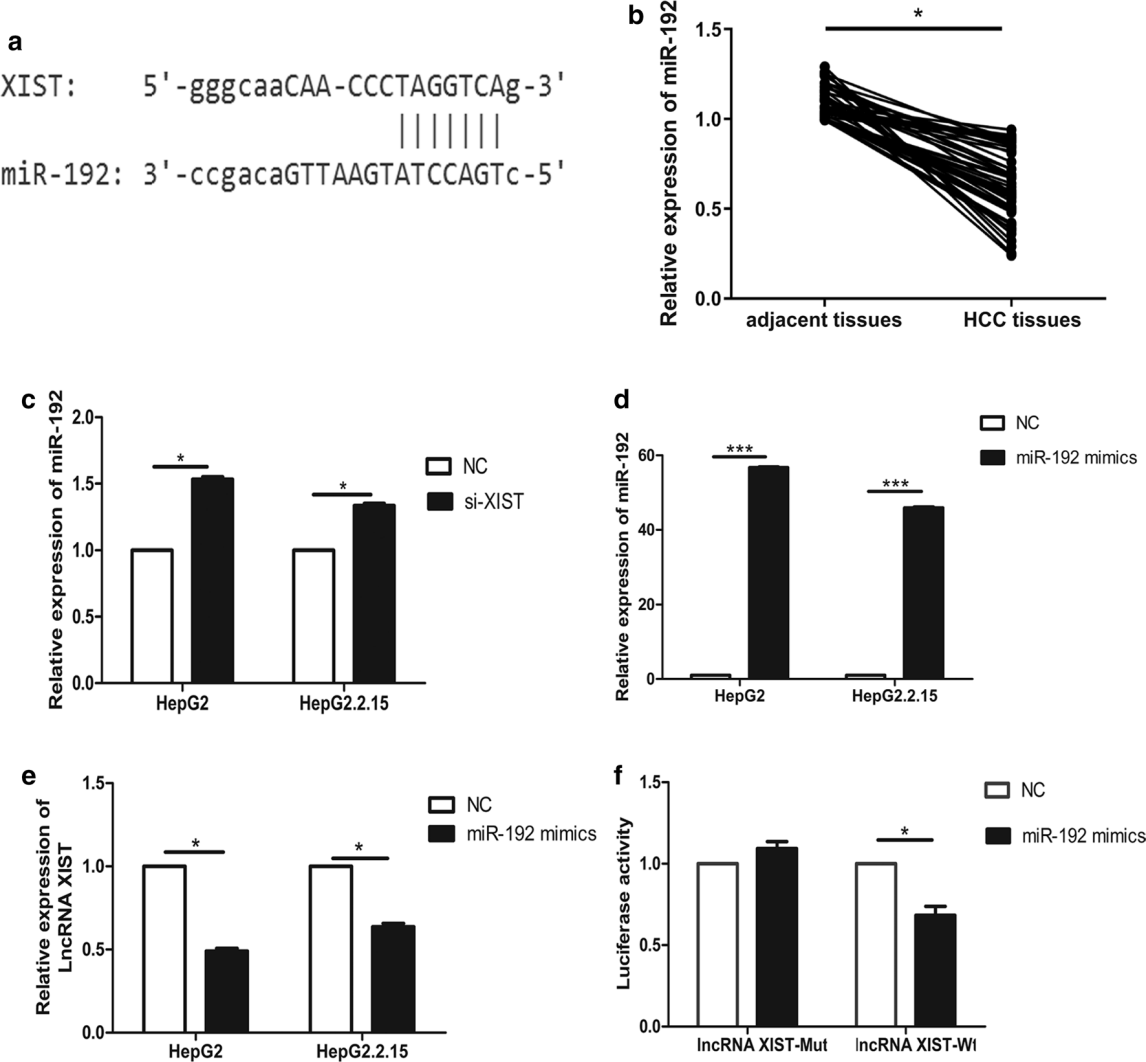
Identification of miR-192 as a target gene of lncRNA XIST. **a** Schematic diagram of the potential binding site between miR-192 and lncRNA XIST. **b** Determination of the mRNA expression of miR-192 in HCC and adjacent tissues. **c** Detection of mRNA expression of miR-192 in cells transfected with si-XIST or NC. **d**, **e** Detection of mRNA expressions miR-192 (**d**) and lncRNA XIST (**e**) in cells transfected with miR-192 mimic or NC. **f** Detection of luciferase activity in HepG2.2.15 cells co-transfected with lncRNA XIST-Mut or lncRNA XIST-Wt and miR-192 mimic or NC. All above the experiments were repeated 3 times *P < 0.05, **P < 0.01, ***P < 0.001

Bioinformatic analysis using StarBase software found that lncRNA XIST had potential binding sites for miR-192 (Fig. 2a). In addition, qRT-PCR results showed that compared with the adjacent tissues, the expression of miR-192 in HCC tissues was observably down-regulated (1.53-fold, *P* < 0.05) (Fig. 2b). Furthermore, si-XIST was transfected into HepG2.2.15 and HepG2 to evaluate the effect of silencing of lncRNA si-XIST on the expression of miR-192. The results indicated that the expression of miR-192 in both types of cell transfected with si-XIST were remarkably up-regulated compared with that in the control groups (*P* < 0.05) (Fig. 2c). Moreover, after transfection with miR-192 mimic, the expression of miR-192 was up-regulated in both types of cell but lncRNA XIST was remarkably down-regulated compared with that in the control groups (*P* < 0.05) (Fig. 2d, e). These results suggested that there was a negative correlation between the expression of miR-192 and lncRNA XIST. Next, lncRNA XIST-Mut or lncRNA XIST-Wt were co-transfected with miR-192 mimic or NC into HepG2.2.15 cells. Luciferase reporter gene assay showed that after co-transfection with miR-192 mimic, the luciferase activity in lncRNA XIST-wt group was observably decreased compared with that in the control group (*P* < 0.05) (Fig. 2e), while there was no significant difference between lncRNA XIST-mut and the control group (*P* > 0.05). These results verified that miR-192 was a direct target gene of lncRNA XIST.

### Effects of lncRNA XIST and miR-192 on HCC cell proliferation and migration

**Fig. 3:**
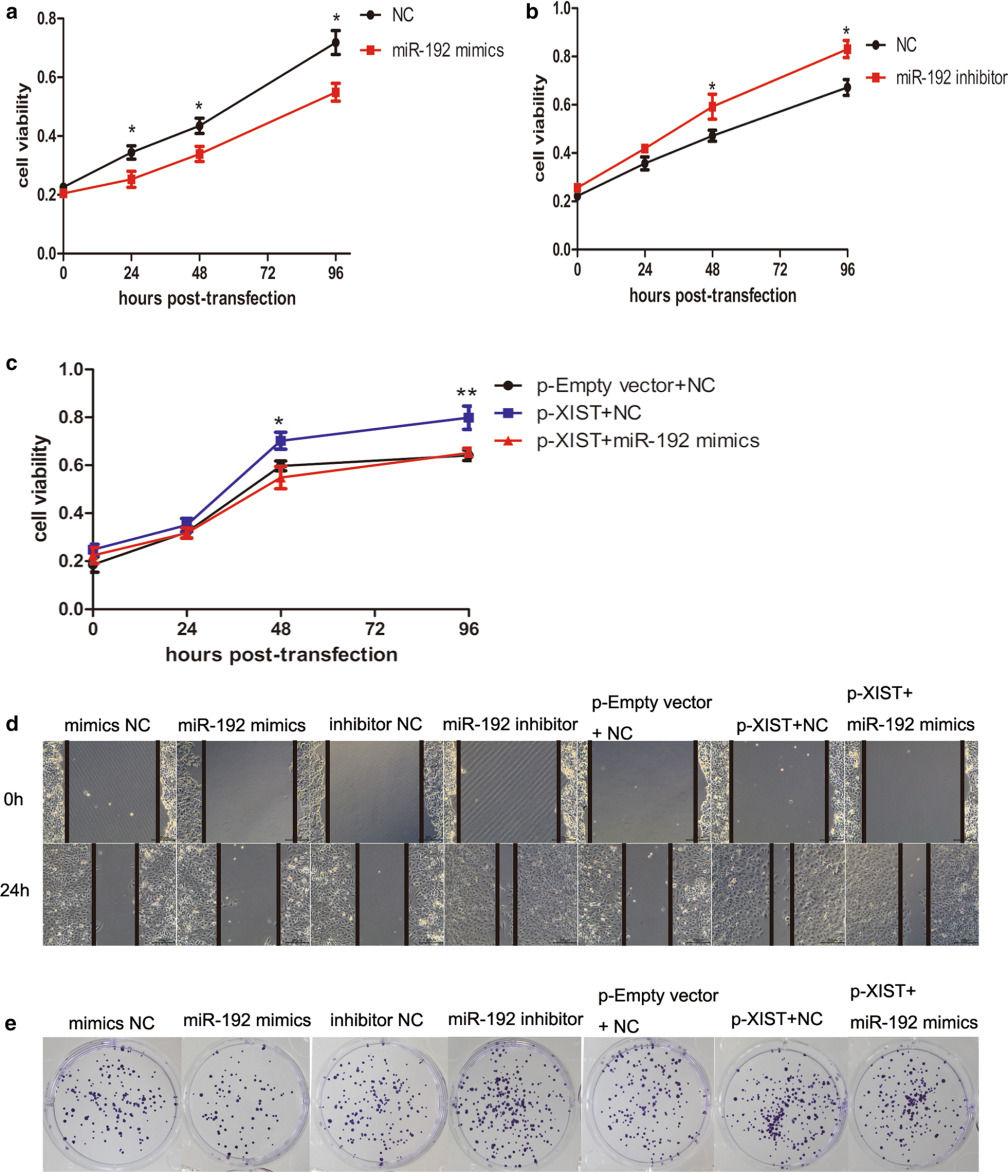
Effects of lncRNA XIST and miR-192 on HCC cell proliferation and migration. **a**–**c** Detection of the proliferation activity of HepG2.2.15 cells by CCK-8 assay. **d**, **e** Determination of migration (**d**) and colony formation (**e**) ability of HepG2.2.15 cells. All above the experiments were repeated 3 times *P < 0.05, **P < 0.01, ***P < 0.001

The miR-192 mimic or miR-192 inhibitor was transfected into HepG2.2.15 cells. CCK-8 assay showed that the proliferation activity of HepG2.2.15 cells in the miR-192 mimic group was remarkably lower than that in the control group (*P* < 0.05) (Fig. 3a), while the proliferation activity of HepG2.2.15 cells in the miR-192 inhibitor group was observably higher than that in the control group (*P* < 0.05) (Fig. 3b). In addition, p-XIST plasmid and/or miR-192 mimic were transfected into HepG2.2.15 cells. It was found that the proliferation activity of HepG2.2.15 cells transfected with p-XIST plasmid was markedly higher than that of the control group (*P* < 0.05, *P* < 0.01) (Fig. 3c), while the proliferation activity of HepG2.2.15 cells in p-XIST + miR-192 mimic group was close to that in control group (*P* > 0.05) (Fig. 3c). These results confirmed that lncRNA XIST could promote the proliferation of HepG2.2.15 cells by targeting and binding to miR-192. Meanwhile, wound healing and colony formation assays showed that overexpression of miR-192 observably inhibited the migration and colony formation of HepG2.2.15 cells, while miR-192 inhibitor showed the opposite effects (Fig. 3d, e). Simultaneously, the migration and colony formation ability of p-XIST + miR-192 mimic were remarkably lower than those of p-XIST group (Fig. 3d, e). These results suggested that lncRNA XIST could enhance the migration and colony formation ability of HepG2.2.15 cells by targeting and binding to miR-192.

### Identification of TRIM25 as a target gene of miR-192

**Fig. 4:**
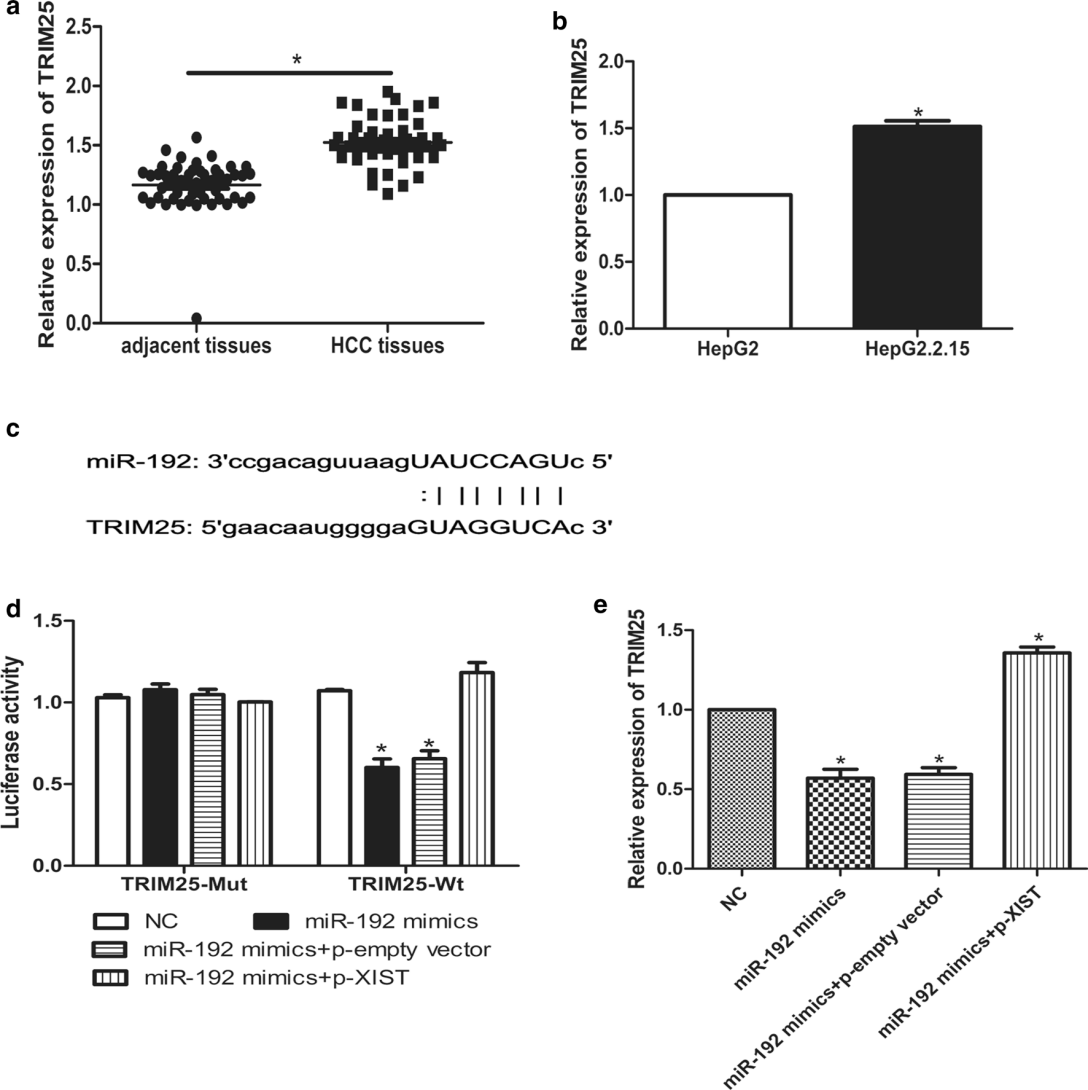
Identification of TRIM25 as a target gene of miR-192. **a**, **b** Detection of the mRNA expression of TRIM25 in HCC and adjacent tissues (**a**) and cells (**b**). **c** Schematic diagram of potential binding sites between TRIM25 and miR-192. **d** Detection of luciferase activity in HepG2.2.15 cells transfected by TRIM25-Mut or TRIM25-Wt with miR-192 mimic or NC. **e** Detection of TRIM25 expression in miR-192 mimic and/or p-XIST transfected HepG2.2.15 cells. All above the experiments were repeated 3 times *P < 0.05, **P < 0.01, ***P < 0.001

Studies have found that TRIM25 is associated with gastric cancer cell metastasis (Zhu et al. 2016). In this study, the expression of TRIM25 in HCC tissues and cells were first determined by qRT-PCR. The results showed that the mean expression levels of TRIM25 in HCC tissues were 1.2 times than that in adjacent tissues (*P* < 0.05) (Fig. 4a), and the expression levels of TRIM25 in HepG2.2.15 cells were also significantly higher than that in HepG2 cells (*P* < 0.05) (Fig. 4b), indicating that TRIM25 was highly expressed in HCC tissues and cells. Next, bioinformatics analysis using the microRNA.org software predicted the potential binding sites of miR-192 in TRIM25 (Fig. 4c). In addition, luciferase reporter gene assay results showed that the luciferase activity of cells in TRIM25-Wt + miR-192 mimic was observably lower than that of the control group (*P* < 0.05) (Fig. 4d), and p-XIST significantly reversed this inhibitory effect. This confirmed that TRIM25 was a direct target gene of miR-192, and lncRNA XIST could inhibit the regulation of miR-192 to TRIM25 by targeting miR-192. Besides, qRT-PCR results found that the expression levels of TRIM25 in the miR-192 mimic group were markedly lower than that of the control group (*P* < 0.05) (Fig. 4e), while the expression of TRIM25 in the p-XIST + miR-192 mimic group was observably up-regulated compared with that in the control group (*P* < 0.05) (Fig. 4e). Taken together, lncRNA XIST could up-regulate the expression of TRIM25 by targeting and binding to miR-192.

## Discussion

As one of the common malignant tumors, HCC has a high morbidity and mortality, and a high postoperative recurrence rate and poor prognosis (Bosetti et al. 2014). Chronic HBV infection is a major risk factor for HCC and can accelerate the development of HCC (Levrero and Zucman-Rossi 2016). Lnc RNA XIST was the major regulator for X chromosome activation in mammals (Yan et al. 2019). Many retrospective clinical studies revealed that lnc RNA XIST may correlate with clinical pathological parameters and predict survival outcomes in cancer patients (Ma et al. 2017). LncRNA XIST has been shown to be abnormally expressed in various tumor tissues including hepatocellular carcinoma, non-small cell lung cancer and malignant glioma (Wei et al. 2017; Zhang et al. 2017). It was reported that lncRNA XIST was highly expressed in HCC cell lines and tissues, and enhanced the cell viability of HCC cells by regulating the expression of miR-139-5p and PDK1 (Mo et al. 2017). Another study reported that lncRNA XIST was highly expressed in HCC tissues and could inhibit the expression of MAPK1 in HCC cells by targeting and binding to miR‐194‐5p, thus accelerating the transfer process of HCC (Kong et al. 2018). However, the role of lncRNA XIST in HBV-related HCC is unknown. In this study, the expression of lncRNA XIST in HBV-related HCC tissues and cells was detected. Our results showed that lncRNA XIST was upregulated in HBV-related HCC tissues and cells, which is consistent with the findings in previous studies (Mo et al. 2017). Therefore, lncRNA XIST is possibly related to the progress of HBV-related HCC. Studies have confirmed that lncRNA can act as a molecular sponge for ceRNA to absorb miRNA, thus inhibiting the regulation of miRNA on target genes (Ballantyne et al. 2016). MiR-192 is downregulated in tumor tissues such as liver cancer, renal cell carcinoma and colon cancer, and has a tumor suppressive effect (Wu et al. 2016; Xu and Fan 2015). LncRNA HOTTIP was found to promote the occurrence and development of HCC by directly binding to miR-192 (Ge et al. 2015). LncRNA XIST promoted the expression of target gene PTEN of miR-181a by targeting and binding to miR-181a, thus accelerating the development process of HCC (Chang et al. 2017).The directly targeting relationship between LncRNA XIST and miR-192 was reported in previous study, but the relationship was still not clear in HCC cells (Gu et al. 2019). Our qRT-PCR results found that there was a negative correlation between the expression of miR-192 and lncRNA XIST. In addition, luciferase reporter assay results confirmed that miR-192 was a direct target gene of lncRNA XIST. Studies have found that HCC cell metastasis is mainly dependent on the proliferation, invasion and migration of tumor cells (Liu et al. 2015). In this study we found that overexpression of miR-192 could remarkably inhibit the proliferation, migration and colony formation ability of HepG2.2.15 cells, while overexpression of p-XIST showed an opposite effect. It was reported that lncRNA FAL1 could induce the migration and proliferation of HCC cells by binding to miR-1236 (Li et al. 2018a). Thus, the results of this study suggested that lncRNA XIST could enhance the proliferation and migration ability of HBV-infected HepG2.2.15 cells by targeting and binding to miR-192.

Mature miRNAs can degrade mRNA by completely or incompletely binding to the 3′-UTR region of mRNA (Rong et al. 2017). It has been shown that TRIM25 as a transcription factor involved in the progression of many tumor diseases (Qin et al. 2016). Zhu et al*.* reported that TRIM25 could promote the occurrence and development of gastric cancer by regulating the TGF-β signaling pathway (Zhu et al. 2016). Overexpression of TRIM25 could enhance the cell viability of prostate cancer cells by regulating the p53 signaling pathway (Takayama et al. 2018). While the role of TRIM25 in HCC is unclear, the opposite conclusions were reported in previous study (Zang et al. 2017; Liu et al. 2020). Therefore, it is reasonable to speculate that the function of TRIM25 in HCC may vary in different cells or histologic subtypes. TRIM 25 has been targeted by many miRNAs in previous studies (Wang et al. 2019a, b; Zhang et al. 2020). But these miRNAs can not explain the contradictory role of TRIM25 in HCC cells. Furthermore, a cell type specific upstream regulator forTRIM25 is the critical to identify its role in HCC. MiR-192 just merits this character. In this study, TRIM25 was upregulated in HCC tissues and cells. Moreover, we also found that the luciferase activity of cells in the TRIM25-Wt + miR-192 mimic group was observably lower than that of the control group, while p-XIST markedly reversed this inhibitory effect. Our qRT-PCR results showed that the expression levels of TRIM25 in miR-192 mimic group were remarkably lower than that of the control group, while p-XIST showed an opposite effect. It was found that IGF2BP3 increased the expression levels of TRIM25 by inhibiting the regulation of TRIM25 by miR-3614, thereby improving the proliferation of breast cancer cells (Wang et al. 2019a, b). Therefore, our results suggested that TRIM25 was a direct target gene of miR-192, and lncRNA XIST could up-regulate the expression of TRIM25 by targeting and binding to miR-192.

Here we reported a new regulated axis for HBV related hepatocellular carcinoma, namely, the lncRNA XIST**-**miR-192**/**TRIM25 axis. Since miR-192, which is correlated with CSC, is identified as the target of lncRNA XIST in HCC cells. Compared with study by Mo et al. the lncRNA XIST**-**miR-192**/**TRIM25 axis may be closer correlated with certain HCC subtypes. Furthermore, this mechanism may provide a premise for exploring the function of TRIM25 in HCC progression.

## Conclusion

In conclusion, the present study assessed the molecular mechanism of lncRNA XIST in HBV-related HCC. These results confirmed that lncRNA XIST could up-regulate the expression of TRIM25 by targeting and binding to miR-192, thus accelerating the occurrence and development of HBV-related HCC, which provided certain theoretical basis for targeted treatment of HBV-related HCC.

## Data Availability

The analyzed data sets generated during the study are available from the corresponding author on reasonable request.
